# Improving Cardiovascular Health in Rural United States: Role of Academic Medical Centers

**DOI:** 10.1016/j.jacadv.2024.100950

**Published:** 2024-05-01

**Authors:** Navin Rajagopalan, Steve W. Leung, Rebecca S. Craft, Alison L. Bailey

**Affiliations:** a Division of Cardiology, University of Kentucky, Lexington, Kentucky, USA; b Center for Heart, Lung and Vascular Health, Parkridge Medical Center, Chattanooga, Tennessee, USA

**Keywords:** rural health, heart failure, cardiovascular imaging, telemedicine

**Figure:**
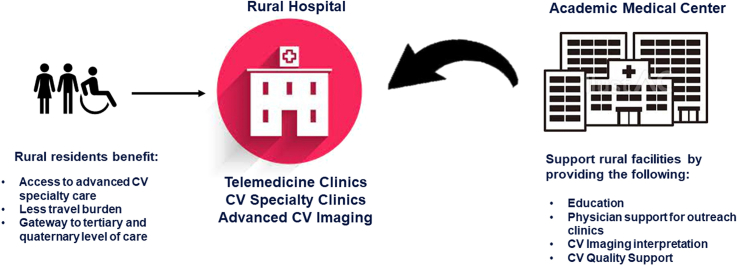

Higher mortality from cardiovascular (CV) disease has been observed in the United States in rural areas compared to urban areas. Approximately 15 to 20% of United States residents live in a rural setting based on county-level data, but <10% of all physicians practice in rural areas.^1^ Rural residents face numerous challenges when accessing medical care, including increased travel distance, higher incidence of CV disease risk factors, and higher rates of poverty. Rural hospitals and health systems are challenged to care for this population because of difficulty recruiting cardiologists, accessing resources to implement new technologies, and maintaining financial stability due to declining reimbursement which can lead to reduction in services or hospital closure. These factors have contributed to the exacerbation of rural-urban health disparities observed over recent decades.^2^

Comprehensive health systems, including academic medical centers (AMCs), collaborate with smaller community hospitals to establish referral networks for patients with complex disease. While this may allow patients with severe acute illness to be seen in tertiary care facilities, many rural patients lack the resources for travel to AMCs for longitudinal outpatient disease management. In our view, AMCs should support the CV programs in their referral networks to improve the overall health of the communities they serve. The focus should not solely be on the referral of patients but also on providing community hospitals with education, training, and clinical support to ensure patients can receive optimal CV care locally when possible. In this article, we explore opportunities for AMCs to interact with rural health facilities as 1 strategy to address rural-urban disparities of care (Figure 1). Although this article focuses on AMCs, the strategies outlined apply to major health systems with comprehensive CV programs. Given the uneven geographic distribution of AMCs across the United States, large health systems and other stakeholders will need to employ these strategies to reduce the urban/rural health care divide.^3^

**Figure 1:**
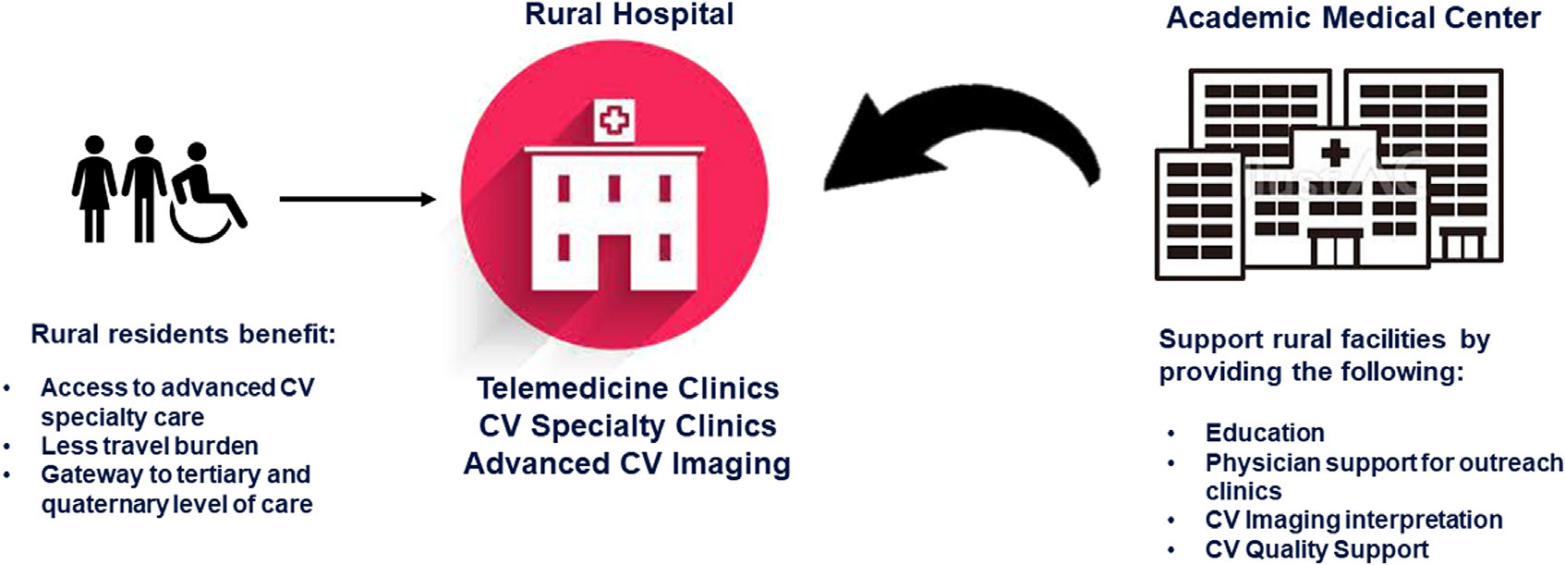
**Academic Medical Centers Can Use Their Resources to Help Rural Hospitals Enhance Their Cardiovascular Programs, Allowing Rural Residents to Have the Same Access to Care As Urban Residents** CV = cardiovascular.

## Telemedicine and subspecialty outreach clinics

Telemedicine enables patients to connect with clinicians without driving to a physical location, an attractive option for rural patients with long travel distances. Telemedicine offers the potential for rural residents to receive specialized care from cardiologists and other subspecialists that would otherwise not be available. AMCs and other major health systems have the resources to collaborate with rural health systems to implement telemedicine on a wider scale than each could do individually. A rural hospital may employ general cardiologists but seeks assistance from an AMC for electrophysiology or heart failure (HF) expertise. Telemedicine-guided optimization of medical therapy for HF could improve health care delivery without requiring travel by either the patient or the cardiologist.^4^

Unfortunately, those individuals who may benefit the most from telemedicine frequently lack the resources to take advantage of this technology. Rural access to broadband internet is suboptimal, a particularly severe problem in Appalachia.^5^ Widespread investment in infrastructure beyond the scope of AMCs is needed to reduce this disparity and take advantage of telemedicine’s benefits. One strategy to mitigate this barrier is for patients to utilize telemedicine not at home, but at their primary physician’s office where they virtually connect to specialists. We recently implemented this model at our institution with a primary care clinic in rural Kentucky without a local cardiologist on site. Although requiring the patient to leave their home, this model offers several advantages, including the opportunity to partner with local offices to assess vital signs, verify medications, and ensure patients understand the care plan described by the cardiologist. Telemedicine partnerships such as this between local health systems and AMCs can hopefully expand in the future and improve CV care delivery.

Management of complex disease may require in-person assessment, as opposed to telemedicine. Outreach clinics provide the opportunity for rural residents to be seen by a cardiologist close to home, thereby reducing travel burden on patients. In regions where general cardiology care is available locally, there is often an unmet need for subspecialty support such as electrophysiology or structural heart disease. Although a procedural intervention such as transcatheter aortic valve implantation may require travel to an urban center, preprocedure testing and specialist consultation could occur locally. For patients with limited financial resources, this could ease the burden and should increase the number of patients who undergo the procedure indicated. Major health systems outside of AMCs have resources to establish outreach clinics, but there are 2 subspecialties which are predominantly part of AMC CV programs: adult congenital heart disease (ACHD) and advanced HF (AHF). This type of specialized care is not often found in rural environments, given the majority of ACHD and AHF specialists work in AMCs or large health systems. One analysis showed that distance from an ACHD center was associated with a decreased probability of ACHD-specific follow-up, but more utilization of health care resources due to increased hospitalizations.^6^ By establishing community-based ACHD outreach clinics, AMCs ensure that these patients, who are often “lost to follow-up,” get appropriate management and receive referrals to a main center when surgical or percutaneous interventions are necessary.

Similarly, AHF specialists are typically employed by centers offering heart transplantation or left ventricular assist devices, which are often located in urban areas and are limited in number.^7^ Rural communities are likely to experience worsening HF burden due to the aging population, high prevalence of HF risk factors, and frequency of multiple comorbidities. Rural HF clinics are needed to manage this patient population, which likely requires multidisciplinary team members in addition to cardiologists. The goal of HF rural outreach clinics should be to optimize the medical management of HF patients, decrease morbidity, and assist rural physicians and care teams with difficult to manage patients, and not solely to identify patients who may benefit from specialized procedures or surgeries. Candidates for left ventricular assist device and transplantation constitute a very small subset of patients that can be seen in outreach clinics and identifying candidates should not be the primary focus.

HF programs at AMCs can provide support to rural hospitals in several additional ways: educational support for the practicing clinicians and pharmacists, sharing of medical therapy protocols and patient education materials, and regular feedback on patients seen by both institutions. At our program, we regularly host rural care teams on-site to visit our HF clinic and meet with team members to foster collaboration and education. Improving overall HF management by empowering and educating the local physicians and team members should be the ultimate goal. AMCs with well-developed HF programs are in a position to use their expertise and experience to help rural health systems with this challenging population that is increasing in number and complexity.

## Advanced CV imaging support

Advanced CV imaging encompasses modalities such as cardiac magnetic resonance imaging (CMR) and coronary computed tomography angiography (CCTA). Current guidelines incorporate CCTA as a Class 1 recommendation for patients with cardiac chest pain without known coronary artery disease.^8^ Advances in cardiac imaging have the potential to increase the disparity between urban and rural health care, whereby the diagnostic algorithm for a common clinical problem, such as chest pain, is determined by the patient’s physical location. For those hospitals seeking to incorporate advanced imaging, AMCs can provide guidance on appropriate scanners and imaging software and can share protocols and best practices. Nurses and technologists at the community hospital can shadow at the AMC’s imaging center to reinforce these concepts and obtain additional education. Patient selection is a key aspect of a successful partnership between the AMC and rural hospitals. There may be imaging indications for CMR that are best performed at a large center, such as complex congenital heart disease and characterization of myocardial masses.

Hospitals need to ensure that advanced cardiac imaging studies are interpreted properly. The number of physicians that have appropriate training in CMR and CCTA interpretation is low. Collaboration between rural hospitals and AMCs can allow imaging studies to be performed locally, with the imaging interpretation occurring at the AMC. Physician recruitment is often challenging in rural hospitals and this collaboration may allow a rural hospital to start an advanced imaging program while the hospital searches for their own imaging specialist(s). In addition, a rural hospital may have a single imaging cardiologist but needs assistance to provide coverage in their absence. A strong collaboration will provide the hospital with feedback regarding imaging quality, and as the relationship progresses, more complex imaging cases could be handled at the rural hospital. AMCs need to have sufficient physicians to provide timely interpretation and resources to provide education to staff of rural hospitals. Several billing models are available; at our AMC, we bill the facility a fee for each study interpretation, while the facility bills the patient’s insurance company directly.

## Programmatic support

AMCs have administrative and nursing leaders who can serve as valuable resources for rural hospitals seeking to advance their CV program and improve program quality. In our experience, this is of particular interest to many rural hospitals as local leaders responsible for quality may have limited experience with CV quality metrics. AMCs can support rural hospitals with developing quality improvement projects and providing assistance with CV accreditation and registries. Encouraging accreditation offered by American College of Cardiology or Joint Commission can indicate the commitment of a hospital to assessing and improving CV quality metrics.

When rural hospitals begin new clinical programs, AMCs can provide support through various initiatives, including peer-review to ensure the delivery of high-quality care; best practice discussions with subject matter experts at the AMC; and the sharing of protocols, policies, workflows, and order sets. AMCs can also support the educational needs of rural hospitals. At our hospital, we have hosted rural hospitals at our cardiac rehabilitation program to share best practices, offered hands-on sonographer training for technicians from rural hospitals, conducted intra-aortic balloon pump training for nurses, and designed a training curriculum for new CV nurses.

Our article is a call to action for AMCs and large health systems to collaborate with rural hospitals to improve the CV health of rural residents, which continues to lag behind urban residents.^1^ AMCs and health systems have the resources and clinicians to assist these facilities with the development and improvement of CV programs. Improving rural health requires major political and societal shifts to decrease an ever-increasing gap in health equity. AMCs and health systems can help take the lead to ensure that rural residents have similar access to advanced CV medicine as their urban counterparts.

## Funding support and author disclosures

Dr Rajagopalan has served as a consultant for Abbott Laboratories. Dr Bailey has served as a consultant for OptumRx and Novo Nordisk. All other authors have reported that they have no relationships relevant to the contents of this paper to disclose.
